# Processing Pipeline for Atlas-Based Imaging Data Analysis of Structural and Functional Mouse Brain MRI (AIDAmri)

**DOI:** 10.3389/fninf.2019.00042

**Published:** 2019-06-04

**Authors:** Niklas Pallast, Michael Diedenhofen, Stefan Blaschke, Frederique Wieters, Dirk Wiedermann, Mathias Hoehn, Gereon R. Fink, Markus Aswendt

**Affiliations:** ^1^Department of Neurology, Faculty of Medicine and University Hospital Cologne, University of Cologne, Cologne, Germany; ^2^In-vivo-NMR Laboratory, Max Planck Institute for Metabolism Research, Cologne, Germany; ^3^Cognitive Neuroscience, Institute of Neuroscience and Medicine (INM-3), Research Center Juelich, Juelich, Germany

**Keywords:** processing pipeline, MRI, atlas registration, stroke, preclinical neuroimaging

## Abstract

Magnetic resonance imaging (MRI) is a key technology in multimodal animal studies of brain connectivity and disease pathology. *In vivo* MRI provides non-invasive, whole brain macroscopic images containing structural and functional information, thereby complementing invasive *in vivo* high-resolution microscopy and *ex vivo* molecular techniques. Brain mapping, the correlation of corresponding regions between multiple brains in a standard brain atlas system, is widely used in human MRI. For small animal MRI, however, there is no scientific consensus on pre-processing strategies and atlas-based neuroinformatics. Thus, it remains difficult to compare and validate results from different pre-clinical studies which were processed using custom-made code or individual adjustments of clinical MRI software and without a standard brain reference atlas. Here, we describe AIDAmri, a novel Atlas-based Imaging Data Analysis pipeline to process structural and functional mouse brain data including anatomical MRI, fiber tracking using diffusion tensor imaging (DTI) and functional connectivity analysis using resting-state functional MRI (rs-fMRI). The AIDAmri pipeline includes automated pre-processing steps, such as raw data conversion, skull-stripping and bias-field correction as well as image registration with the Allen Mouse Brain Reference Atlas (ARA). Following a modular structure developed in Python scripting language, the pipeline integrates established and newly developed algorithms. Each processing step was optimized for efficient data processing requiring minimal user-input and user programming skills. The raw data is analyzed and results transferred to the ARA coordinate system in order to allow an efficient and highly-accurate region-based analysis. AIDAmri is intended to fill the gap of a missing open-access and cross-platform toolbox for the most relevant mouse brain MRI sequences thereby facilitating data processing in large cohorts and multi-center studies.

## Introduction

Understanding brain function in health and disease at different hierarchical levels requires collaborative interdisciplinary efforts using multiple experimental methods. Neuroimaging, especially magnetic resonance imaging (MRI), is a critical element of that approach since the use of MRI preserves the anatomical morphology of the brain tissue almost perfectly. Conscious of the high data integrity, large-scale human MRI initiatives are currently underway to provide standardized sharing repositories (Hodge et al., 2016; Gorgolewski et al., 2017) and processing tools (Rex et al., 2003; Jenkinson et al., 2012). In order to be able to compare information derived from different studies, images are spatially normalized to a common coordinate system such as the brain atlas with defined coordinates and assigned structures from Talairach and Tournoux (Fang et al., 1995) or the Montreal Neurological Institute/International Consortium of Brain Mapping (MNI/ICBM; Mazziotta et al., 1995). In order to achieve similar routine atlas-based neuroinformatics of mouse brain MRI, several challenges need to be overcome: (1) the image signal-to-noise ratio (SNR) is dramatically reduced due to image voxels in mice which are 10–15-fold smaller in all dimensions (Nieman et al., 2005); (2) scanner hardware consisting of gradients, coils as well as the animal fixation and anesthesia need to be miniaturized and adapted to the mouse body and physiology (Driehuys et al., 2008); (3) human MRI processing tools usually do not work with mouse brain data due to the striking differences in voxel size; and (4) a common 3D MRI-compatible brain atlas with a detailed segmentation is needed to facilitate atlas-based neuroinformatics at different scales. Recent developments in scanner hardware, e.g., ultra-high-field MRI scanner (>7T) and dedicated ultra-sensitive coils, enabled *in vivo* mouse brain MRI with structural anatomical details at 100 μm in-plane resolution as well as brain-wide network analysis at the functional and structural level (Hoehn and Aswendt, 2013). However, there is currently no standardization or consensus on MRI acquisition, processing, and atlas-based neuroinformatics. Although several mouse brain atlases have been developed and applied (Hess et al., 2018), not all of them are continuously updated and maintained to be accessible online. The most detailed 3D mouse brain atlas, the Allen Brain Reference Atlas (ARA), provides more than 1,000 brain structures (Lein et al., 2007; Dong, 2008). However, the ARA was generated from two-photon microscopy images with a very low image correlation to MRI (e.g., ventricles appear black and not white as in T2-weighted MRI). Most labs rely on custom-made code or adapt their data to the processing requirements of human imaging toolboxes (van Meer et al., 2010; Hübner et al., 2017; Green et al., 2018), often with lack of validation. Existing software pipelines (Supplementary Table S1) require commercial software, use different MRI atlases or do not incorporate algorithms for both, structural and functional MRI (Budin et al., 2013; Koch et al., 2019). The associated lack of reproducibility and comparability represents a key drawback for reliable multi-center and translational animal studies. Therefore, we developed a novel the Atlas-based Imaging Data Analysis Pipeline, AIDAmri, for structural and functional MRI of the mouse brain using the ARA coordinate system. AIDAmri provides an automated, efficient and highly accurate region-based analysis of multi-parametric MRI, such as anatomical T2-weighted MRI, diffusion tensor imaging (DTI) and resting-state functional MRI (rs-fMRI). The modular and open-source concept was developed in Python 3.6 for cross-platform use. That allows the critical comparison of different imaging methods and studies. Each processing step of the pipeline was validated with qualitative and quantitative measures on mouse brain MRI data acquired at 7.0, 9.4 and 11.7T using different mouse strains and experimental stroke models. Stroke was chosen as an example, as lesions result in dynamic brain deformations due to tissue swelling and atrophy, which presents a major challenge for all automated processing and atlas registration algorithms.

## Materials and Methods

### Pipeline Overview

**Figure 1:**
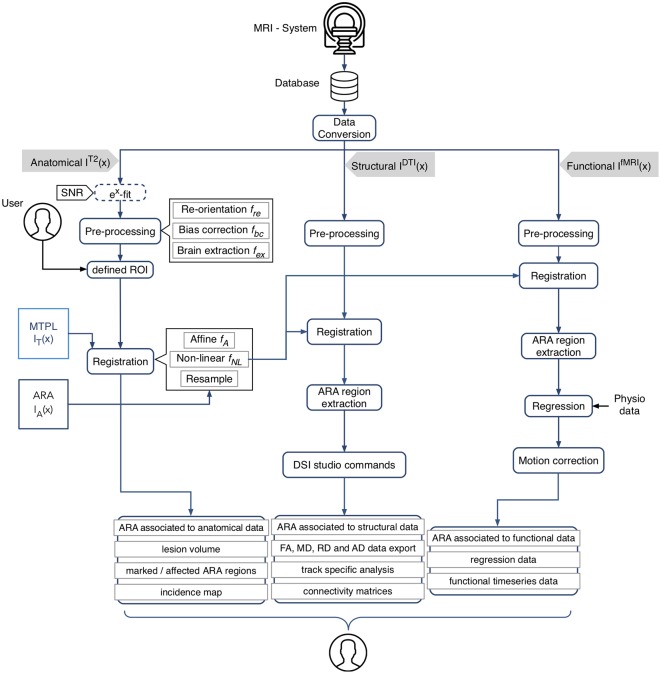
Schematic overview of AIDAmri processing modules and subsequent computational steps for anatomical data (T2-weighted and T2 map), structural (diffusion tensor imaging, DTI) and functional data (resting-state functional magnetic resonance imaging, rs-fMRI). The given image function *I*(*x*) represents the 3D MRI image space and describes all intensities at the position $x=\{\vec{x},\vec{y},\vec{z}\}$. All data types are pre-processed using a re-orientation *f*_re_(*x*), bias correction *f*_bc_(*x*) and brain extraction *f*_ex_(*x*). The user has the opportunity to define individual regions of interest (ROIs), e.g., a lesion mask, to compare particular areas over different measurements by generating an incidence map. The combined transformation *f* of the affine *f*_A_ and non-linear transformation *f*_NL_ is applied to MRI template MTPL *I*_T_(*x*) and subsequently the ARA *I*_A_(*x*) with the pre-processed data set *I*^T2^(*x*). DTI *I*^DTI^(*x*) and rs-fMRI *I*^fMRI^(*x*) processing steps were implemented based on established protocols (Budde and Song, 2010; Kim et al., 2012; Gorges et al., 2017). AIDAmri generates a variety of outputs such as the connectivity matrices which can be used for further atlas-based connectivity analysis. Icons designed by Smashicons from www.flaticon.com.

The AIDAmri pipeline enables the processing and analysis of both structural and functional mouse brain MRI through distinct modules which can also be used separately. In the following, we provide a detailed explanation of the processing steps (Figure 1). The software pipeline is freely available on Github^1^. For a detailed how-to and installation instructions see the manual (Supplementary Material, Manual). The AIDAmri interface (GUI) is available for executing the main functions.

A reference adult mouse T2-weighted (T2-w), DTI and rs-fMRI data set acquired at 9.4T is available for testing purposes^2^. Image processing is performed in the Allen Mouse Common Coordinate Framework (CCF v3) using the Allen Mouse Brain Reference Atlas, ARA^3^. It is possible to use manually drawn regions-of-interest (ROIs) or other brain atlases as well. Here, the ARA was implemented as it is the most advanced brain atlas to-date (Supplementary Figure S1 and Supplementary Table S2). To describe the following complex morphological operators (e.g., the image registration), we chose the commonly used mathematical model to describe the image with the given image function *I*(*x*) where *x* describes all voxel positions with $x=\{\vec{x},\vec{y},\vec{z}\}$. Based on that model, the given functions transfer voxels of one subset *X* into another subset *Y* with *f*(*x*) = *x* ∈ *X* | *f* (*x*) ∈ *Y*} in the three-dimensional image space.

We have included algorithms for the most widely used and most relevant MRI sequences assessing structural and functional connectivity changes using MRI which are not available in other pipelines (see Supplementary Table S1, for a selection of other mouse brain imaging pipelines):

(1) T2-weighted MRI (acquired with Turbo spin echo (TSE) or Rapid Acquisition with Refocused Echoes (RARE) sequences) for high-contrast and high spatial resolution imaging of brain anatomy and pathophysiology (e.g., hyperintense signal for segmentation of stroke lesions),
(2) Quantitative T2-mapping (measured for example by multi slice multi echo, MSME, sequences), e.g., for longitudinal monitoring of contrast agent accumulation or lesion development,
(3) DTI, which maps the diffusion process of the water molecules in biological tissues (acquired with diffusion-sensitized sequences such as echoplanar imaging, EPI, along at least 6 directions). DTI is used to derive quantitative measures such as Fractional Anisotropy (FA), Mean Diffusivity (MD), Radial Diffusivity (RD), and Axial Diffusivity (AD). These measures relate to biological differences and are used for clinical diagnosis (Bihan et al., 2001). Furthermore, MRI-based tractography using DTI, provides non-destructive, 3D, brain-wide connectivity maps, which are used in animal and human studies too (Budde and Song, 2010),
(4) Resting state functional MRI (rs-fMRI), which provides functional data on temporal correlation of spontaneous blood-oxygenation level-dependent (BOLD) changes at rest that reflect regional interactions between two particular brain regions in task-negative state. Functional connectivity derived from rs-fMRI is used in preclinical and clinical studies (Grefkes and Fink, 2014; Gorges et al., 2017).

### MRI Data Acquisition

The MRI data was acquired at the Max Planck Institute for Metabolism Research, Cologne, using a 94/20USR BioSpec Bruker system (Bruker, BioSpin, Ettlingen, Germany) equipped with a cryo-coil and operated with ParaVision (v6.0.1). The mice were anesthetized initially with Isoflurane (2%–3% in 70/30 N_2_/O_2_) and head-fixed in an animal carrier using tooth and ear bars. Fixation and anesthesia are necessary to minimize movement artifacts. Respiration, and body temperature were noninvasively monitored using an MR-compatible monitoring system (Small Animal Instruments Inc., New York, NY, USA) and displayed and recorded using a custom-made data acquisition system based on DASYLab (measX, Mönchengladbach, Germany). To maintain body temperature at 37°C, a feedback-controlled water circulation system (medres, Cologne, Germany) was used. T2-weighted, rs-fMRI and DTI scans (Table 1) were sequentially acquired using *n* = 22 C57BL6/J mice which received photothrombotic stroke in contrast to sham surgery as described previously (Toda et al., 2014). The animal experimental data were collected and managed using a custom-made and cloud-based relational animal database^4^ described in detail elsewhere (Pallast et al., 2018). Also, *N*_T_ = 40 test data sets linked to previously published (Aswendt et al., 2012; Green et al., 2018) or unpublished (provided by Mathias Hoehn) projects. The data sets were acquired at different field strengths and with animals of different strains.

**Table 1:** Characteristics of the performed 9.4 T MRT measurements.

Scan type	T_R_^4^ (ms)	T_E_^5^ (ms)	Acq. time (s)	Matrix size	Resolution (mm)	FOV^6^ (mm)	Flip angle
T2w^1^	5,500	32.5	352	256 × 256 × 48	0.068 × 0.068 × 0.3	17.5 × 17.5	90°
rs-fMRI^2^	1,420	18	149	128 × 128 × 20	0.141 × 0.141 × 0.4	18.0 × 18.0	90°
DTI^3^	3,000	17.5	840	128 × 128 × 20	0.141 × 0.141 × 0.4	18.0 × 18.0	90°

*Over 100 data sets with the given properties were used to test AIDAmri. Abbreviations: ^1^T2-weighted MRI, ^2^resting-state functional MRI, ^3^Diffusion Tensor Imaging, ^4^repetition time, ^5^echo time, ^6^field of view*.

The pipeline AIDAmri processes DTI and rs-fMRI data independently, but it is necessary to acquire an anatomical reference image in the same measurement, such as a T2-weighted image.

#### Step 1–Data Conversion and Signal-to-Noise Calculation

In the first step, Bruker raw data are converted to the commonly used format of the Neuroimaging Informatics Technology Initiative (NIfTI; Cox et al., 2004). Other imaging formats, such as DICOM, need to be converted including all header information (e.g., using the software MRIcron^5^ or the Python package dicom2nifti^6^). The AIDAmri converter algorithm automatically detects the type of performed measurement and applies conversion in the correct order by reading the respective image header. According to that information, the converted NIfTI-files are sorted in related folders. The anatomical dataset is used to calculate the nonlinear registration which is later applied to the structural and functional data. AIDAmri not only transforms T2-weighted images from the raw data but also calculates the exponential decay over the echo time from multi-echo sequences to calculate quantitative T2 maps.

Automated quality control is included based on SNR calculations based on the automatic noise variance estimation which was chosen proven to be more precise in human MRI (Brummer et al., 1993). Furthermore, that method is less error-prone as the common approach to calculate the SNR (Henkelman, 1985), by placing a ROI inside anatomical regions and another ROI in the noise, and calculate the ratio of the mean signal and the standard-deviation as SNR (Levenberg, 1944).

#### Step 2–Pre-processing

##### Image Re-orientation

All subsequent steps, especially the atlas registration, depend on a defined image orientation of the input data. According to the common three-dimensional coordinate system with three planes, we decided to implement the right-hand “neurological” RAS system. In our setting, the mouse lies prone and is inserted with the head-first into the scanner. Images were acquired selecting “head-supine” in ParaVision. Hence, a transformation *f*_re_(*x*) is necessary to re-orientate the images in standard space. This results in images viewed from feet-to-head direction and the right side of the mouse is on the right side of the image.

##### Bias-Field Correction and Brain Extraction

**Figure 2:**
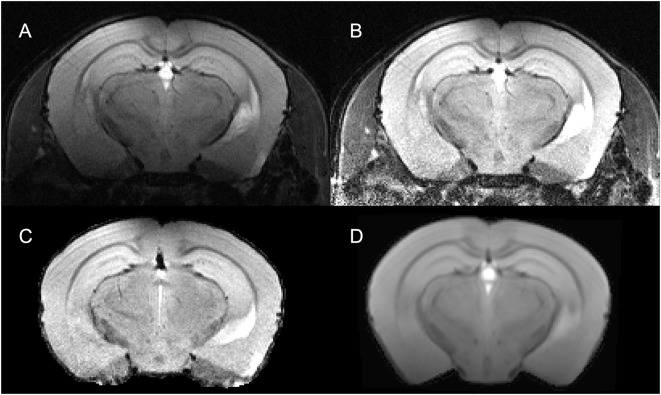
Visualization of step 2—pre-processing for a representative T2-weighted data set. The raw data set *I*^T^^2^(*x*) **(A)** underwent a re-orientation *f*_re_(*x*) and bias field correction using multiplicative intrinsic component optimization (MICO) *f*_bc_(*x*) to reduce inhomogeneities **(B)**. The subsequent registration is done on a brain extracted volume $I_{\text{ex}}^{T2}(x)$ **(C)** by deforming the MTPL *I*_T_(*x*) **(D)** with affine *f*_A_(*x*) and non-linear *f*_NL_(*x*) transformation.

In case of surface coils, there is a strong bias field on the MR image (Figure 2A). AIDAmri contains an automated bias-field correction *f*_bc_(*x*). We implemented the multiplicative intrinsic component optimization (MICO) which was previously used only in human MRI (Li et al., 2014). We compared MICO to the widely-used N4 bias-field correction (Tustison et al., 2010). A total of *n* = 22 T2-weighted (T2-w) data sets and 10 DTI data sets were compared using the coefficient of variations (CV) metric (see “Results” section) leading to full integration of MICO. The corrected images (Figure 2B) are used to apply the brain extraction (skull stripping). AIDAmri runs the FMRIB Software Library (FSL) tool BET with the options -r set to the brain radius in mm and -R for an “robust” iterative estimation of the brain center. Thus, MR images with variable center-of-gravity from animals positioned slightly different between scans will not affect the skull stripping accuracy (Figure 2C; Smith, 2002). To allow FSL to process the data, the data dimension of need to be scaled by a factor of 10 to simulate human-similar voxel sizes. In order to avoid image interpolation, up- and downscaling is carried out automatically only for the related NIfTI header file, whereas the voxel size of the raw image remains the same.

##### Region-of-Interest Segmentation

The user then has the option to define ROIs. We use that option to delineate the ischemic stroke lesion on T2-weighted images using the 3D snake evolution tool of ITK-SNAP^7^ (Yushkevich et al., 2006). The resulting segmentation is used to evaluate specific areas separately by generating a list of regions that are overlaid with the segmented area of the brain, e.g., to proof the position of an electrode. If several segmented ROIs are provided, a color-coded incidence map can be created, e.g., to highlight how many mice had a certain brain area affected by the stroke.

##### Mouse Brain Atlas and MRI Template

**Figure 3:**
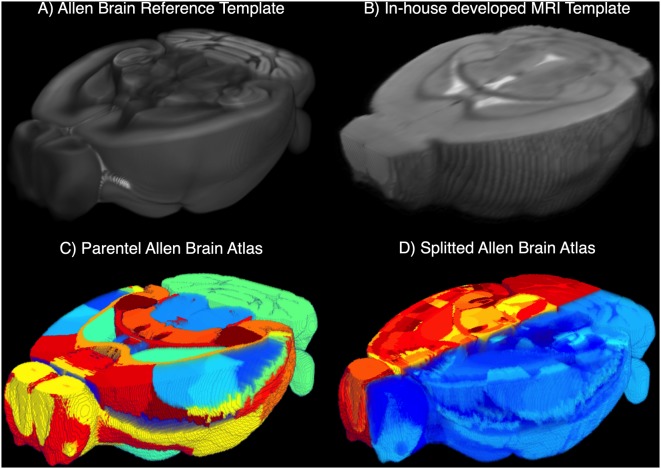
3D cut-outs of the **(A)** Allen Brain Reference Atlas (ARA) and **(B)** the in-house developed MRI template (MTPL). The annotations of the ARA *I*_A_(*x*) and the related ARA template **(A)** are overlaid with the MTPL *I*_T_(*x*) consisting of *N* = 30 T2w. Parental ARA labels *I*_A↑_(*x*) **(C)** and detailed ARA labels with hemisphere split **(D)**.

We developed an in-house MRI template (MTPL) *I*_T_(*x*) with strong correlation to the T2 raw images *I*^T^^2^ (see Supplementary Figure S2) by using *N* = 30 randomly chosen data sets of healthy C57BL6 mice of similar age. The mean of all voxels described in the gray matter (GM), white matter (WM) and cerebrospinal fluid (CSF) were calculated over all *N*, and the resulting template was associated with the original ARA (Allen Brain Reference Atlas, CCF v3, 50 μm isotropic resolution; Figures 3A,B). To obtain a complete overview of the ARA label IDs, we transferred the available information about label IDs, acronyms and names to a custom-made relational database (https://github.com/maswendt/AIDAdb; to access the file, a www.ninoxdb.de account is required). The database lists all brain regions according to the atlas ontology and provides simple access to associated parent and child labels. Using that database, we selected hierarchical lower regions of interest and defined the related parent labels (Figure 3C) to build a parental atlas *I*_A↑_(*x*). This results in a reduction from >1,000 regions in *I*_A_(*x*) to 49 regions in *I*_A↑_(*x*). In order to compare regions of the left vs. right hemisphere, the original ARA and the custom parental ARA were we splitted along the sagittal plane (Figure 3D).

##### Registration

**Figure 4:**
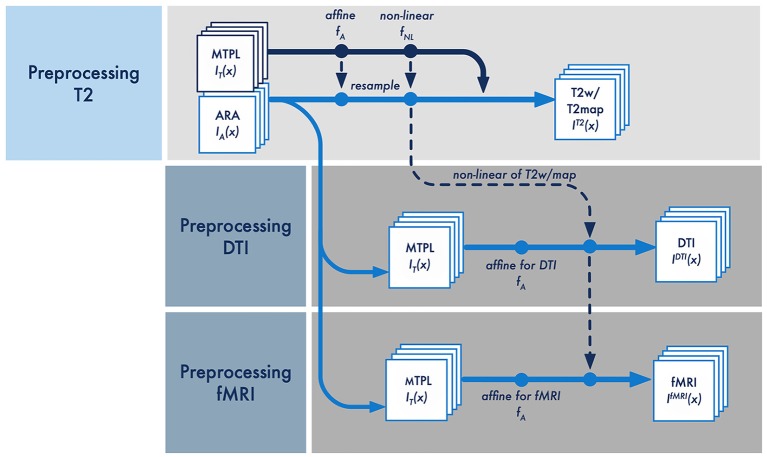
Schematic overview of the multi-step registration procedure for the T2-weighted, DTI and rs-fMRI data [*I*^T^^2^(*x*), *I*^DTI^(*x*) and *I*^fMRI^(*x*)]. The affine *f*_A_ and non-linear transformation *f*_NL_ is applied to MRI template (MTPL) *I*_T_(*x*) and subsequently the ARA *I*_A_(*x*) with the pre-processed data set *I*^T^^2^(*x*). The non-linear deformation *f*_NL_ between MTPL and the T2w/T2m is calculated only once and then linked to the respective affine transformation to pre-processed data of DTI *I*^DTI^(*x*) and rs-fMRI *I*^fMRI^(*x*).

We decided to use a specific multi-step registration scheme (Figure 4). The initial assumption of AIDAmri is that all given information of the ARA *I*_A_(*x*) is represented in the reference image space *X*. The assignment of this information to the individual MRI measurements *I*^T^^2^(*x*), *I*^DTI^(*x*) and *I*^fMRI^(*x*) is achieved by a suitable transformation *f* which transforms *X* in the acquisition image space *Y*, such that

f:X→Y

Each individual transformation *f* is a combination of an affine *f*_A_(*x*) and non-linear *f*_NL_(*x*) transformation computed using NiftyReg (Centre of Medical Image Computing, University College London, UK). NiftyReg was chosen based on a direct comparison (see Figure 2D and Supplementary Figure S2) of registration accuracy with the developed MTPL *I*_T_(*x*) to FSL (Jenkinson et al., 2012), Advanced Normalization Tools (ANTs; Avants et al., 2008) and elastiX (Klein et al., 2010). Consequently, for linear affine registration the symmetric global block matching approach was implemented [NiftyReg, reg_aladin (Modat et al., 2014) with 6 degrees-of-freedom (DOF)]. To describe non-linear deformations, landmark points are placed on the reference image and iteratively deformed [NiftyReg, reg_f3d (Modat et al., 2010), with 12 DOF]. The non-linear transformation *f*_NL_(*x*) describes subcortical brain changes, such as a baseline shift. The multi-step registration requires the different scans to be orientated the same, which can be achieved by copying the orientation from the first to the subsequent scan(s). In that scenario, the non-linear deformations do not change significantly over different scans of one imaging session. Hence, the quantification of *f*_NL_(*x*) is only necessary once and the relative change can be applied to all data sets that are acquired in one session (Figure 4). The differences between each data set in one section can be adequately described by an affine *f*_A_(*x*) transformation which includes scaling, rotation, translation, compression and shearing. The registration procedure exclusively serves the purpose to transfer data of ARA to the related MRI data sets and to correlate functional and structural data. The processing steps to extract the connectivity information from DTI and activity information from rs-fMRI are conducted with the unmodified raw-data.

The deformation *f* between *I*^T^^2^(*x*) and *I*_T_(*x*) is quantified minimizing the Kullback–Leibler divergence (Figure 4; Kullback and Leibler, 1951). The combined transformation *f* of the affine *f*_A_(*x*) and non-linear transformation *f*_NL_(*x*) are applied to the MTPL *I*_T_(*x*) and subsequently the ARA *I*_A_(*x*) with the pre-processed data set *I*^T^^2^(*x*). Both, the affine transformation *f*_A_(*x*) and the non-linear transformation *f*_NL_(*x*) are stored for each processed data set separately. As an important factor influencing registration precision, we set the Jacobian determinate penalty to 0.3 where the user can increase the minimum deformation field from 1 mm to 5 mm depending on the strength of the required deformation. The affine transformation *f*_A_(*x*) is quantified by minimizing the Kullback–Leibler divergence between the current DTI or fMRI measurement and the related T2 measurement *I*^T^^2^(*x*). At this processing step, we have an ARA for all assumed data sets *I*^T^^2^(*x*), *I*^DTI^(*x*) and *I*^fMRI^(*x*), which lies in the same image space and is completely superimposed with the respective data. All subsequent fully automatic analysis steps of functional and structural data are based on a quantification that are provided by the anatomical regions of the associated ARA.

In order to validate the performance of the automated registration, we compared the automatically transformed ARA template of *N*_T_ = 40 test data sets (Table 2) with an ARA template that was semi-automatically registered by two independent observers O1 and O2 using a previously described landmark-based registration approach with the help of the software 3DSlicer^8^ (Kikinis et al., 2014; Ito et al., 2018). The error range between the transformations of both observers was set as a reference. We calculated the distance between *I*_A_(*x*) and the ARA templates of both approaches to find out where a high agreement exists. The Euclidian distance or *L*^2^-Norm were used as one of the most common mathematical quantity of the distance between two-dimensional image functions. However, a slight shift or a rotation would hardly change the appearance of the image and possibly not be detectable by the human viewer at all. To avoid any dependency on changes in intensity the normalized cross-correlation (CrC) has been established (Avants et al., 2008). Since, the correlation between image fidelity and image quality is in some cases insufficient (Silverstein and Farrell, 1996), we also applied the Structural Similarity Index (SSIM; Wang et al., 2004) to end up with a satisfactory quality description. The idea of structural information is that pixels have strong interdependencies especially if they are spatially close. With these three metrices, we quantified the overall characteristic of the human perception to detect distortion between two images.

**Table 2:** Data overview of *N*_T_ = 40 data used to validate the registration.

Name	Scanner (T)	Scans (Σ)	Type	Matrix size	Resolution (mm)	Animals
Data set 1	11.7	14	T2m	256 × 256 × 16	0.068 × 0.068 × 0.6	Nu/Nu	Adult nude mice
Data set 2	11.7	5	T2m	256 × 256 × 12	0.068 × 0.068 × 0.6	DCX-Luc	Adult transgenic DCX-Luc mice
Data set 3	9.4	10	T2w	256 × 256 × 48	0.068 × 0.068 × 0.6	C57BL6/N	Adult wildtype mice
Data set 4	7.0	11	T2w	128 × 128 × 30	0.109 × 0.109 × 0.5	C57BL6/N	Adult wildtype mice with stroke

*All data were acquired on scanners with different characteristics or different image geometries. The registration of the listed data was compared with a manually slice-wise registration performed by two independent observers*.

#### Step 3–DTI and rs-fMRI Processing

##### Pre-processing and Registration

To correlate all given information of the anatomical information of the *I*^T2^(*x*) to its related DTI *I*^DTI^(*x,t*) or fMRI *I*^fMRI^(*x,t*) measurements, some additional pre-processing steps are necessary. First, the dimension of the data must be reduced from 4D to 3D from *I*(*x*,*t*) to *I*(*x*). For this purpose and to minimize the noise and reduce artifacts, a minimum filter is applied over time and then the resulting three-dimensional data set is filtered with a Gaussian kernel. These filters preserve structures necessary for a sufficient registration whereas image noise is suppressed. Based on the previously mentioned assumption, for the registration of *I*^DTI^(*x*) or fMRI *I*^fMRI^(*x*) only an affine transformation *f*_A_(*x*) is performed and the non-linear transformation *f*_NL_(*x*) is applied from the previous T2 calculation (Figure 4). Subsequent DTI and rs-fMRI processing steps were implemented based on established protocols, which led to valid results in previous studies (Budde and Song, 2010; Kim et al., 2012; Gorges et al., 2017).

##### DTI—Structural Connectivity

Motion artifacts in diffusion imaging mostly origin from subtle head movements due to the fast breathing rhythm, which results in repetitive voxel displacements in the x-y plane. To quantify and spatially correct anatomical dissimilarities with 6 degrees of freedom (DOF), we apply a slice-wise motion correction using FSL MCFLIRT (Jenkinson et al., 2002). Unfortunately, MCFLIRT co-registers every volume in a time series to the one volume in the midst of the series to detect slow physical movement. By adapting the correction from a volume based to a slice-based mode of operation, AIDAmri splits each data set into slices, correcting them separately and merging the motion corrected slice series back into one 4D data set. The motion-corrected data are then fed into DSI-Studio (Yeh et al., 2013). The non-brain tissue was discarded by applying a binary mask of the brain extraction to the original DTI data set *I*^DTI^(*x*, *t*). The data are reconstructed within DSI-Studio, based on an electrostatically optimized protocol of Jones30 (Skare et al., 2000) with 30 gradient directions. The reconstructed diffusion images are used to perform fiber tracking and analyze the data with respect to the associated regions of the ARA. All reconstructed data sets, AD, radial (RD), MD and the fraction anisotropy (FA) are being exported separately.

The whole brain tractography is conducted with the deterministic streamline propagation using Euler’s methods (Basser et al., 2000) and terminates if a total fiber number of one million fibers is reached. The tracking starts from a random voxel position and propagated with a step size of 0.5 mm. All fibers shorter than 0.5 mm or longer than 12 mm were discarded, whereas the tracking is terminated if the angle between two consecutive directions exceeds 55°. The fiber termination criteria were previously tested on several data sets with healthy animals for best parameter settings, concerning true and false fiber generation. The analysis provides connectivity matrices, in which the rows and columns of the matrices represent a region of the ARA and the entries display the connectivity strength between two particular regions.

##### rs-fMRI—Functional Connectivity

Before the regional characteristics can be evaluated by means of rs-fMRI, some optimizations need to be implemented. The mouse in our setup is fixed with ear bars and a tooth bar minimizing head movements during acquisition. Nevertheless, spontaneous excitement due to fluctuations in anesthesia phases and respiratory motion may affect image stability. Therefore, we recorded the breathing during the measurement to identify regressors describing respiratory artifacts. The physiological data were sampled during EPI data acquisition, indicated by overlaid trigger pulses. The pre-processing of the breathing signal included the detection of inspiration peaks and baseline correction using the median values. Additionally, slice-wise motion correction is applied to the raw rs-fMRI *I*^fMRI^(*x,t*) by the same approach as for DTI. This additional correction is necessary to detect additional displacement between slices or fast respiratory rhythms. Since for many scientific applications, such as event-triggered fMRI, a slice time correction is essential, it is possible to switch on that function in AIDAmri and perform a correction with FSL SliceTimer (Jenkinson et al., 2002).

Completed by the pre-processed physiological recording, all of this data has been merged into a single multichannel file. The following processing steps were implemented based on the processing steps in FSL FEAT (Woolrich et al., 2001) with some modifications. For example, the smoothing was adapted with a spatial filter. Due to anisotropy of the voxels in z-direction, the spatial filter is applied in the x- and y-plane and not over the whole volume as in FSL FEAT. In our case, the spatial filter smooths the data with FWHM of 3.0 mm and a high-pass filter with a cut-off frequency of 0.01 Hz that reduces additional noise sources. The registered ARA is used to extract the regions in the functional domain generating a 4D file (x, y, slices, region masks) in NIfTI format. That file includes all transformed ARA regions, whereas each three-dimensional region is defined by a binary mask. Among all repetitions of the resting state fMRI data, the mean of the intensities of the voxels of a region is calculated and this average constitutes the averaged time series of the specific region.

## Results

### Bias-Field Correction

**Figure 5:**
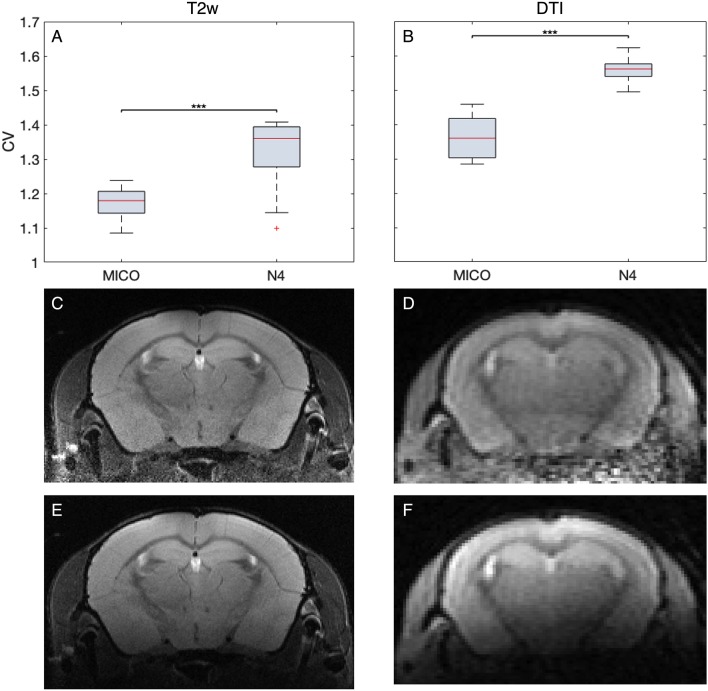
Quantitative and qualitative comparison of MICO and N4 bias-field correction. The calculation of the degree of homogeneity revealed lower coefficient of variations (CV) for MICO compared to N4 for 22 T2w and 10 DTI measurements **(A,B)**. Representative MR images comparing MICO **(C,D)** and N4 **(E,F)** bias-field corrected images for T2w and DTI, respectively.

Magnetic field inhomogeneities induced by insufficient shimming, imperfect coil placement and susceptibility artifacts at tissue borders directly relate to image quality. To measure the bias-field, we tested the N4 against the MICO algorithm (Figure 5). MICO has so far only been tested for human MRI. The comparison was conducted on 22 T2w data sets and 10 DTI data sets with the CV as metric. For both data sets, MICO-based bias-field correction resulted in lower CV values compared to the N4 algorithms (*p* < 0.001) and better corrections of the bias-field.

### Registration

**Figure 6:**
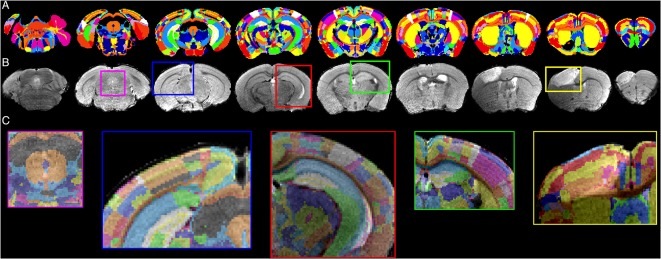
Registration results. Representative transformed ARA annotations **(A)** that are registered on an T2w data set **(B)** with detailed views shown as overlay **(C)**.

**Figure 7:**
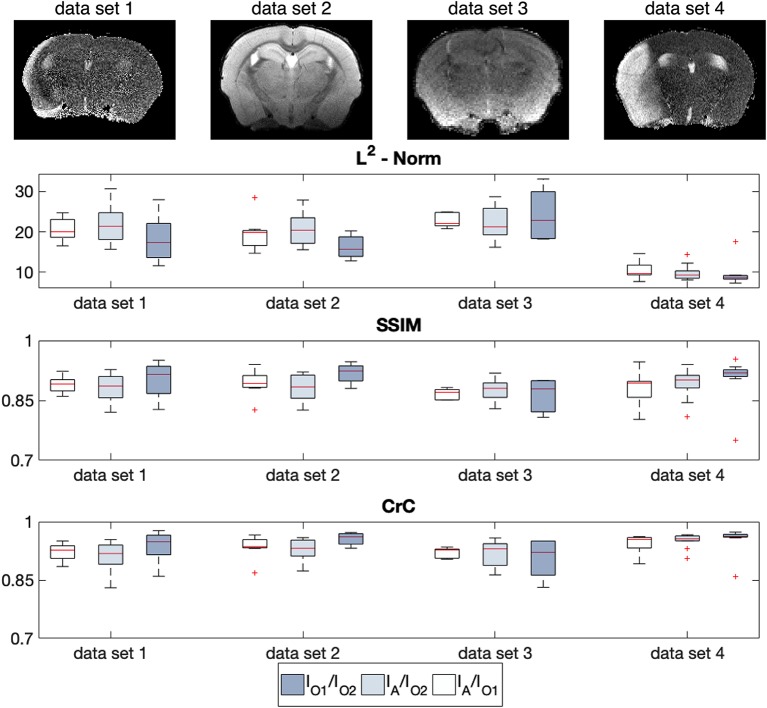
Quantitative registration quality control using a slice-wise comparison of *N*_T_ = 40 imaging data grouped in four sets with three metrics [L2-Norm, Structural Similarity Index (SSIM), Cross-Correlation (CrC)] between AIDAmri *I*_A_ and two observers *I*_01_ and *I*_02_. The different properties of each data sets are listed in Table 2 and one example slice is shown above each evaluation. Whereas, the ground-truth was determined with the error range of *I*_01_/*I*_02_, the average error between the automated approach of AIDAmri and the observer-dependent approach *I*_A_/*I*_01_ and *I*_A_/*I*_02_ showed no significant differences in all three metrices.

The results of the multi-step registration for a representative mouse brain with large stroke-related deformations are shown in Figure 6. The stroke lesion is distinguishable in the anterior slides of T2w data set as the hyperintense regions. Even strong deformations of the anatomical structure are realistically contoured by the algorithm, such that the ARA is precisely overlaid with the T2w data set. In addition to the qualitative assessment, we applied a quantitative quality control (Figure 7) using a slice-wise comparison of *N*_T_ = 40 MR images selected from four different MRI datasets (Table 2). Two experienced observers used a semi-manually landmark-based approach to overlay the atlas.

For each data set the error range between the transformations made by observer 1 and 2 *I*_01_/*I*_02_ was used as reference. Due to the different imaging properties and the subjective landmark placing there was a large variability in the median between both observers. In comparison to the automatic registration of AIDAmri the deviation to the ground truth defined by both observers, minimal and not statistically significantly differences for all quantitative measurements (L2, SSMI, CrC). For example, for data set 3 in Figure 7; the median of the SSMI between both observers *I*_01_/*I*_02_ is 0.878. Compared to that reference value the median of AIDAmri to both Observes is 0.870 for *I*_A_/*I*_01_ and 0.880 for *I*_A_/*I*_02_. In conclusion, the deviation varies only between 0.81 and 0.92 in the SSIM for all evaluated data sets and shows comparable error values for the CrC.

**Figure 8:**
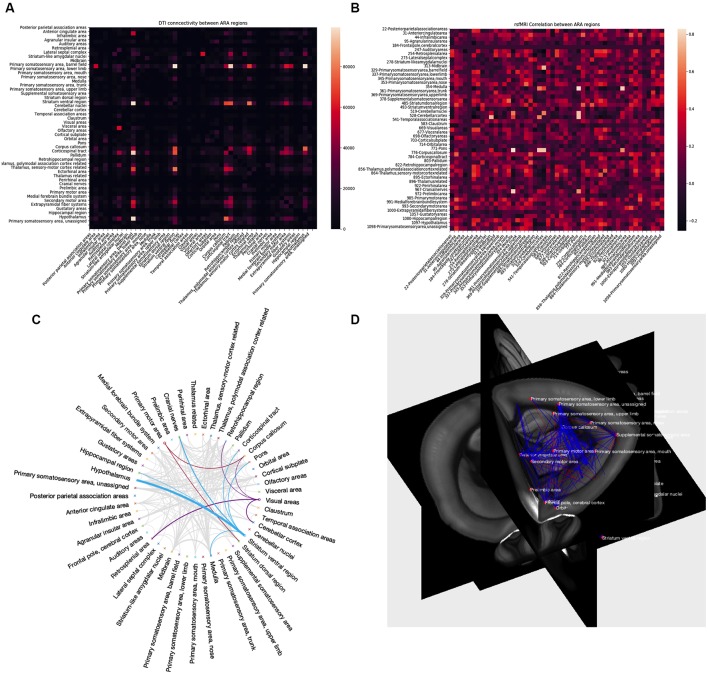
AIDAmri output. Structural and functional information of DTI** (A)** and fMRI **(B)** can be represented as adjacency matrices by using the plot function. The entries in the matrices represent the number of tracks passing or ending in the ARA regions of the DTI and activity pattern of rs-fMRI among all ARA regions to evaluate the results using graph theoretical approaches. Other ways to visualize connectivity patterns (plot function not included in AIDAmri): the circular representation of a row or column vector **(C)** where thicker lines relate to higher matrix values **(C)** and 3D visualization of connectivity in the anatomical context, here the registered atlas **(D)**.

After successful processing with AIDAmri, the results offer various possibilities for further data analysis (Figure 8). Depending on the field of research, users have the opportunity to evaluate their data quantitatively and qualitatively. AIDAmri includes plot functions to visualize structural and functional information of DTI (Figure 8A) and rs-fMRI (Figure 8B) as adjacency matrices. To achieve a more detailed quantitative evaluation a variety of possibilities are freely available and can be used regardless of the processing pipeline. For example, predefined regions can be examined in regard of their structural and functional properties^9^. Relationships between the ARA regions can also be visualized in a circular Graph^10^. The Brain Connectivity Toolbox (Rubinov and Sporns, 2010) can be used for a quantitative evaluation of the DTI data based on graph theory. Likewise, rs-fMRI data can be evaluated with FSLNETS^11^. In each case, no further pre-processing steps are necessary and the output of AIDAmri can be directly fed into the established tools.

## Discussion

Currently, a variety of tools are available for human imaging studies, offering either a full evaluation (Cui et al., 2013) and/or step-by-step workflow (Rubinov and Sporns, 2010). In the pre-clinical environment, standardization of MRI acquisition, processing and sharing standards still need major development. Therefore, the unique translational advantage of MRI, e.g., to directly probe novel scan protocols and biomarker findings from bench to bedside awaits exploitation (Jaiswal, 2015). Here, we present a novel Atlas-based Imaging Data Analysis Pipeline (AIDAmri) for structural and functional MRI of the mouse brain. AIDAmri represents the first region-based processing pipeline, that extracts the structural and functional information from T2w, DTI and rs-fMRI data, and which enables a region-by-region analysis of preclinical MRI data based on the Allen Brain Reference Atlas (ARA). Importantly, the developed MRI template facilitates co-registration of MRI data with the ARA, which would be impossible by a direct registration. Since the template is co-registered with the ARA in the original image space, research groups of other labs can customize the ARA in higher hierarchical levels to map their individual regions-of-interest without the need to downscale the information. For example, we provide both a (hemisphere-splitted) detailed as well as custom-made parental atlas. The parental atlas is particularly useful for the analysis of DTI and rs-fMRI with have intrinsically lower image resolution and are stronger affected by susceptibility artifacts. Although we carefully validated the registration, the striking differences in original image size and resolution between the atlas and the DTI/rs-fMRI can result in pixel interpolations and region-mismatches, e.g for small thalamic nuclei or single cortical layers. In that case, we recommend the parental atlas, with larger brain regions, where interpolations have negligible effects. In comparison to other atlases, the ARA provides not only the most-detailed structural 3D atlas but also access to the Allen Institute cell type, transcriptomics and brain connectivity database (Lein et al., 2007; Oh et al., 2014). AIDAmri was written in Python 3.6 using freely available algorithm tools. The modular structure enables efficient processing and the possibility to modify or add modules. To enhance the comparability to other fields of research and to ensure its applicability to a variety of neurological questions, AIDAmri has been comprehensively tested and optimized by following steps. First, we implemented a novel SNR measurement, which has been shown to outperform manual or other semi-automatic measurements (Sijbers et al., 2007). Second, to prepare the data for registration with the ARA, pre-processing steps including re-orientation, bias-field correction, and brain extraction were implemented. We successfully implemented the MICO bias-field correction, which was applied before only on human data (Li et al., 2014). We could show, that MICO generates significantly better data even in the pre-clinical environment than the well-known N4 algorithm. Finally, we applied a quantitative quality control to verify that the developed multi-step registration process works robustly. In a statistical analysis, the results achieved by two-independent and trained observers were found to be not different from the automated registration for various mouse strains. Registration accuracy was also valid for pathologies such as stroke with significant brain deformation due to, e.g., oedema or necrosis. The AIDAmri output contains functional and structural connectivity matrices for all (selected) ARA regions. These matrices can be used to analyze differences in the brain network between health and disease. For the first time, AIDAmri provides in one common processing pipeline and one common atlas system quantitative structure-function relationships, which are known to be crucial to understand the structural underpinnings of brain function and brain plasticity (Straathof et al., 2019). Future studies may focus on the integration of other imaging modalities, e.g., single photon emission computed tomography (SPECT) or positron emission tomography (PET), to the ARA. AIDAmri contributes to the awareness-raising effort of the scientific community to standardize diverse datatypes and analyses across species (Sejnowski et al., 2014) and will facilitate data processing in large cohorts and multi-center studies.

## Ethics Statement

This study was carried out in accordance with the recommendations of ARRIVE and IMPROVE guidelines (Kilkenny et al., 2010; Percie du Sert et al., 2017). The protocol was approved by the Landesamt für Natur, Umwelt und Verbraucherschutz North Rhine-Westphalia (reference number 84-02.04.2016.A461, 84-02.04.2014.A226 and 84-02.04.2012.A190).

## Author Contributions

NP designed and implemented the pipeline, wrote the manuscript. MD contributed to the code, provided algorithms and processing steps. SB contributed to the code, performed trouble-shooting and testing. FW performed manual atlas registration. DW and MH provided MRI data, contributed to the structure of the pipeline and wrote the manuscript. GF contributed to the design of the pipeline and wrote the manuscript. MA supervised the project, performed atlas registration and wrote the manuscript.

## Conflict of Interest Statement

The authors declare that the research was conducted in the absence of any commercial or financial relationships that could be construed as a potential conflict of interest.

## Footnotes

^1^https://github.com/maswendt/AIDAmri

^2^https://doi.org/10.12751/g-node.70e11f

^3^http://mouse.brain-map.org/static/atlas

^4^https://github.com/maswendt/AIDAdb

^5^https://www.nitrc.org/projects/mricron

^6^https://pypi.org/project/dicom2nifti/

^7^www.itksnap.org

^8^https://www.slicer.org/

^9^https://de.mathworks.com/matlabcentral/fileexchange/27983-slicer

^10^https://github.com/paul-kassebaum-mathworks/circularGraph

^11^www.fmrib.ox.ac.uk/fsl
