# Supplementary material for: Processing Pipeline for Atlas-Based Imaging Data Analysis of Structural and Functional Mouse Brain MRI (AIDAmri)
Source: Front Neuroinform. 2019 Jun 4;13:42. doi: 10.3389/fninf.2019.00042 (PMC6559195; doi:10.3389/fninf.2019.00042)
Supplement: Supplementary file 1 [file Data_Sheet_1.docx]

Supplementary Material

# Supplementary Tables

**Table 1:** Comparison of rodent MRI processing pipelines

| Pipeline | Software | Registration tool | T2w/  T2 map | dMRI | fMRI | Atlas |
| --- | --- | --- | --- | --- | --- | --- |
| AIDAmri  <https://github.com/maswendt/AIDAmri> | [Python](https://www.python.org/)  (open source, cross-platform) | NiftyReg | ✓/✓ | ✓ | ✓ | ARA v3 |
| ANTX  (Koch et al., 2017)  <https://github.com/philippboehmsturm/antx> | [Matlab](https://de.mathworks.com/products/matlab.html)  (commercial, cross-platform) | SPM8  (SPMMouse) and  Elastix | ✓/ |  |  | ARA v3 |
| MouseMorph  Powell et al. (unpublished)  <https://github.com/nmpowell/mousemorph> | [Python](https://www.python.org/)  (open source, cross-platform) | NiftyReg | ✓/ | ✓ |  | 3D MRM atlas |
| (Budin et al., 2013)  No available for downlaod | [Midas server](http://www.midasplatform.org/)  (open source, cross-platform) | 3DSlicer | ✓/ | ✓ |  | 3D MRM atlas |

**Table 2**: Summary of in vivo and ex vivo mouse brain atlases based on MRI or microscopy

| Name | Distributor | Type | Animals | Image resolution | Labels | References |
| --- | --- | --- | --- | --- | --- | --- |
| ARA  (CCF v3) | [The Allen Institute](http://mouse.brain-map.org/), Seattle, USA | Serial two-photon (STP) tomography merged for 3D | 1675 mice, 56-day old C57BL/6J | 10 um^3^ isotropic  800 slices | 1305 (neural structures, fiber tracts and gross anatomical features) | (Ding et al., 2016; Dong, 2008; Lein et al., 2006; Oh et al., 2014) |
| tlas3D* | [Neural Systems and Graphics Computing Laboratory, University of Oslo, Norway](http://rbwb.org/) | Photographs of Nissl and AChE stained cryosections | 26 mice, 3 months old C57BL/6J | 4.5 um/pixel - 132 (coronal), 21 (sagittal) and 30 (horizontal) | 730 (neural structures, fiber tracts and gross anatomical features) | (Hjornevik et al., 2007; Paxinos, 2013) |
| Mouse Brain Library | [The Mouse Brain Library](http://www.mbl.org/atlas232/atlas232_frame.html) | Nissl staining, celloidin-embedded brains | 1 mouse, 294-day old C57BL/6J | 4.5 um/pixel, 30 um thick, 17 sections | none | - |
| BrainMaps | [BrainMaps](http://brainmaps.org/index.php?p=speciesdata&species=mus-musculus), Usrey Lab UC Davis, USA | 2D images of histological stains (e.g. Giemsa, Nissl, AChE and others) | 1 mouse per stain | 0.46x0.46x25 um  8-234 slides (depending on stain) | none | (Mikula et al., 2007) |
| AMBMC | [Australian Mouse Brain Mapping Consortium](http://imaging.org.au/AMBMC/Model) | Ex vivo T1/T2*-w 16.4T MRI | 18 mice, C57BL/6J | 15 um^3^ isotropic  499 slices | 62 (main anatomical structures) | (Janke and Ullmann, 2015) |
| MAP 2003 Atlas | [Laboratory of Neuro Imaging, University of Southern California, Los Angeles, USA](http://www.loni.usc.edu/atlases/Atlas_Detail.php?atlas_id=19) | In vivo T2-w 11.7T MRI and Nissl/myelin stain blockface imaging | 165 C57BL/6J, 100-day old | 60 um^3^ isotropic  256 slices (MRI) and 6.7 um/pixel (histology) | 774 (neural structures, fiber tracts and gross anatomical features) | (MacKenzie‐Graham et al., 2007) |
| MR histology | [Duke University, USA and Biomedical Informatics Research Network (MBIRN)](http://www.civm.duhs.duke.edu/pubs/supplemental/NeuroImage200702/index.html) | Ex vivo T1/T2/-weighted 9.4T Oxford magnet | >100 C57BL/6, 9-12 weeks old | 21.5/43.0 | 39 (gross anatomical features) | (Hawrylycz et al., 2011; Johnson et al., 2010) |
| 3D MRM atlas | [Brookhaven National Laboratory, Upton, USA](https://www.bnl.gov/world/) | In vivo T2-weighted MRI, 9T | C57BL/6 mice, 12-14 week old | 100 um^3^ isotropic | 20 (gross anatomical features) | (Ma et al., 2008) |
|  |  | Ex vivo T2*-weighted 17.6T MRI | 12 weeks, male, C57BL/6J | 47 um^3^ isotropic  256 slices | 20  (gross anatomical features) | (Ma et al., 2005) |

# Supplementary Figures

# Figure 1. Sagittal views of common mouse brain atlases (upper row) and corresponding level of detail of segmented brain regions.


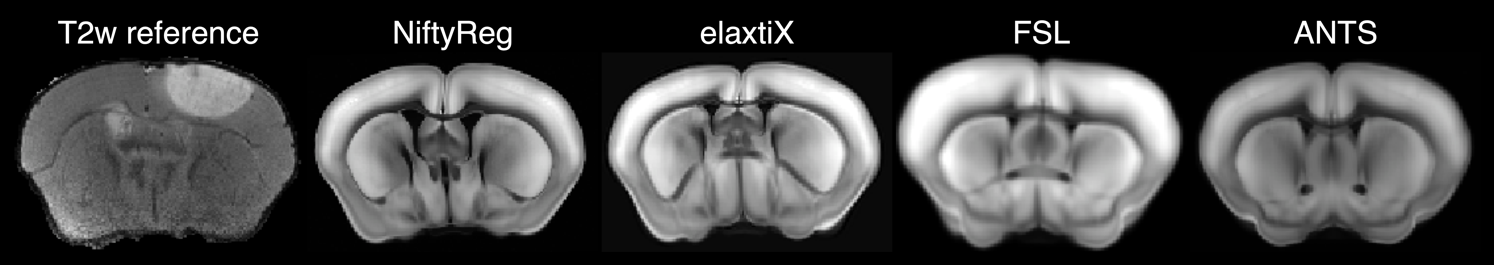


Figure 2. The in-house developed template $\boldsymbol{I}_{\boldsymbol{T}}\left( \boldsymbol{x} \right)$ was registered to a T2w reference test dataset (<https://web.gin.g-node.org/pallastn/AIDA_dataset>) using four different transformation tools with modified parameters: NiftyReg, elastiX, FSL, ANTS. In each case we conducted an affine and non-linear b-spline registration and applied the transformation to the ARA template. In comparison, the ARA template transformed with NiftyReg meets the requirements best.

# References

Budin, F., Hoogstoel, M., Reynolds, P., Grauer, M., O’Leary-Moore, S. K., and Oguz, I. (2013). Fully automated rodent brain MR image processing pipeline on a Midas server: from acquired images to region-based statistics. *Frontiers in Neuroinformatics* 7, 15.

Ding, S., Royall, J. J., nkin, S., Ng, L., Facer, B., Lesnar, P., et al. (2016). Comprehensive cellular‐resolution atlas of the adult human brain. *Journal of Comparative Neurology* 524, 3127–3481.

Dong, H. (2008). The Allen reference atlas: A digital color brain atlas of the C57Bl/6J male mouse. *John Wiley & Sons Inc*.

Hawrylycz, M., Baldock, R. A., Burger, A., Hashikawa, T., Johnson, A. G., Martone, M., et al. (2011). Digital Atlasing and Standardization in the Mouse Brain. *PLoS Computational Biology* 7, e1001065.

Hjornevik, T., Leergaard, T. B., Darine, D., Moldestad, O., le, A., Willoch, F., et al. (2007). Three-dimensional atlas system for mouse and rat brain imaging data. *Frontiers in Neuroinformatics* 1, 4.

Janke, A. L., and Ullmann, J. F. (2015). Robust methods to create ex vivo minimum deformation atlases for brain mapping. *Methods (San Diego, Calif.)* 73, 18–26.

Johnson, G., Badea, A., Brandenburg, J., Cofer, G., Fubara, B., Liu, S., et al. (2010). Waxholm space: an image-based reference for coordinating mouse brain research. *NeuroImage* 53, 365–72.

Koch, S., Mueller, S., Foddis, M., Bienert, T., von Elverfeldt, D., Knab, F., et al. (2017). Atlas registration for edema-corrected MRI lesion volume in mouse stroke models. *Journal of Cerebral Blood Flow & Metabolism*, 0271678X1772663.

Lein, E. S., Hawrylycz, M. J., Ao, N., Ayres, M., Bensinger, A., Bernard, A., et al. (2006). Genome-wide atlas of gene expression in the adult mouse brain. *Nature* 445, 168–176. doi:10.1038/nature05453 .

Ma, Y., Hof, P., Grant, S., Blackband, S., and Neuroscience, B.-R. (2005). A three-dimensional digital atlas database of the adult C57BL/6J mouse brain by magnetic resonance microscopy. *Neuroscience*. doi:10.1016/j.neuroscience.2005.07.014 .

Ma, Y., Smith, D., Hof, P. R., Foerster, B., Hamilton, S., Blackband, S. J., et al. (2008). In vivo 3D digital atlas database of the adult C57BL/6J mouse brain by magnetic resonance microscopy. *Front Neuroanat* 2, 1. doi:10.3389/neuro.05.001.2008 .

MacKenzie‐Graham, A. J., Lee, E., Dinov, I. D., Yuan, H., Jacobs, R. E., and Toga, A. W. (2007). Multimodal, Multidimensional Models of Mouse Brain. *Epilepsia* 48, 75–81. doi:10.1111/j.1528-1167.2007.01244.x .

Mikula, S., Trotts, I., one, J., and Jones, E. G. (2007). Internet-enabled high-resolution brain mapping and virtual microscopy. *NeuroImage* 35, 9–15. doi:10.1016/j.neuroimage.2006.11.053 .

Oh, S., Harris, J. A., Ng, L., Winslow, B., Cain, N., Mihalas, S., et al. (2014). A mesoscale connectome of the mouse brain. *Nature* 508, 207. doi:10.1038/nature13186 .

Paxinos, G. (2013). Paxinos and Franklin’s the mouse brain in stereotaxic coordinates. *Academic Press*.

**
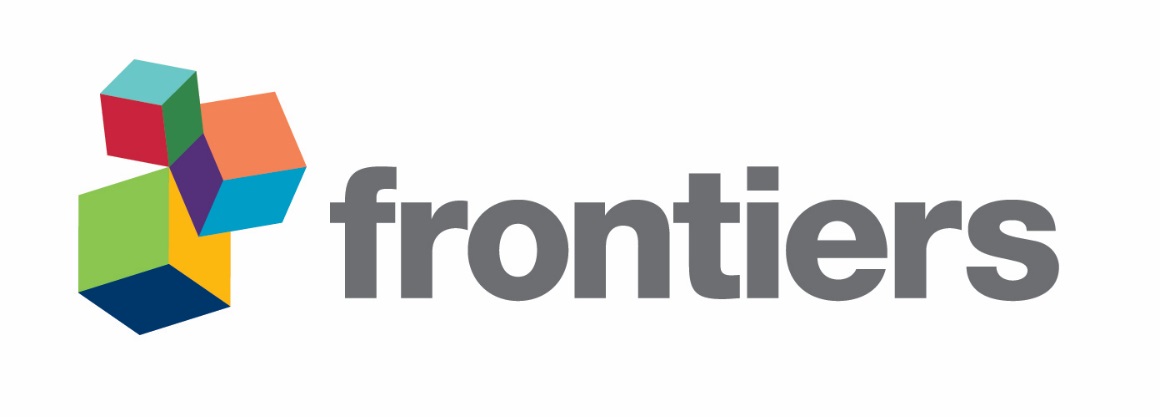
**
