# Supplementary material for: Processing Pipeline for Atlas-Based Imaging Data Analysis of Structural and Functional Mouse Brain MRI (AIDAmri)
Source: Front Neuroinform. 2019 Jun 4;13:42. doi: 10.3389/fninf.2019.00042 (PMC6559195; doi:10.3389/fninf.2019.00042)
Supplement: Supplementary file 2 [file Data_Sheet_2.pdf]

Manual: Atlas - based Processing Pipeline for functional and structural MRI Data AIDAmri

Niklas Pallast
Department of Neurology
University Hospital Cologne

2019

1 Introduction

The Atlas based Processing Pipeline for functional and structural MRI Data (AIDAmri) was developed for automated processing of preclinical high-field magnetic resonance imaging (MRI) data of the mouse brain. AIDA is able to associate structural and functional datasets. That includes T2-weighted MRI (T2w), diffusion weighted MRI or diffusion tensor imaging (DTI) and functional MRI (fMRI). The Allen Brain Reference Atlas (ARA) is registered on each of these MRI datasets and is used to analyse regions of interest. Furthermore, the regions of the ARA are used as seed-points for the connectivity and activity matrices.

2 Installation

1. Download the folders `/bin` and `/lib` by using this link
   `/bin` and `/lib` should be located in the same directory
2. Download & Install DSI-Studio and copy the install path into
   `.../bin/3.2_DTIConnectivity/dsi_studioPath.txt`

3. Download & Install FSL 5.0.1

4. Download & Install Cmake, open the software and click in the upper menu on *Tools* → *How To Install For Command Line Use* like shown in Figure 1 and follow the steps

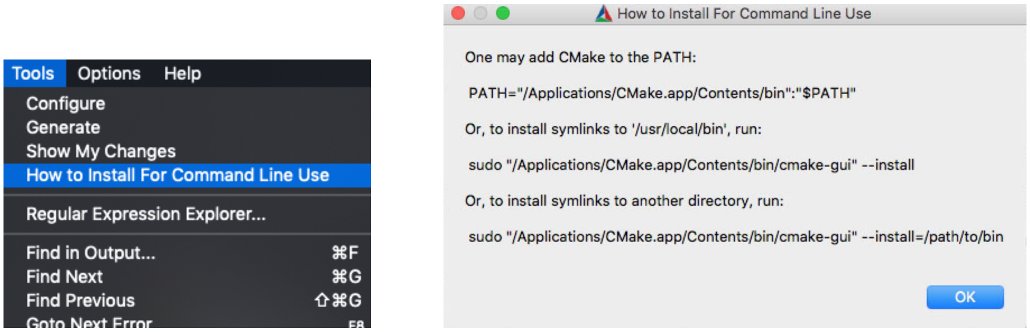

(a) Menu of Cmake software

(b) Install for command line use

Figure 1: Installation instructions to install Cmake

5. Download & Install Python 3.6 or higher using Anaconda and enter the command to install necessary packages
`pip install nipype=1.1.2 lmfit=0.9.11 progressbar2=3.38.0`

6. Install NiftyReg by conducting the following steps:

   a. Generate your source folder `.../NiftyReg/niftyreg_source`

   b. Download NiftyReg from the git by replacing `<path>` by your personal path and enter the following command
   `git clone git://git.code.sf.net/p/niftyreg/git <path>/niftyreg_source`

   c. Change folder by typing in the command windows `cd <path>/niftyreg_source`

   d. Type in the command line
   `git reset --hard 83d8d1182ed4c227ce4764f1fdab3b1797eecd8d`

   e. Follow the steps described here

3 Usage of AIDA

Attention: All program examples are only listed with the mandatory input parameters. For more details, call python `.../python <command> -h`. The command line examples are given with the identifier `testData<No.>.nii.gz` and can be identically applied to other data. The test dataset is freely available and can be downloaded from https://doi.org/10.12751/g-node.70e11f. After a successful download, open the `AIDA_gui.py` in the `/bin` folder to process the test data set. Go to folder and type the following command line `python AIDA_gui.py` to open the window shown in Figure 2

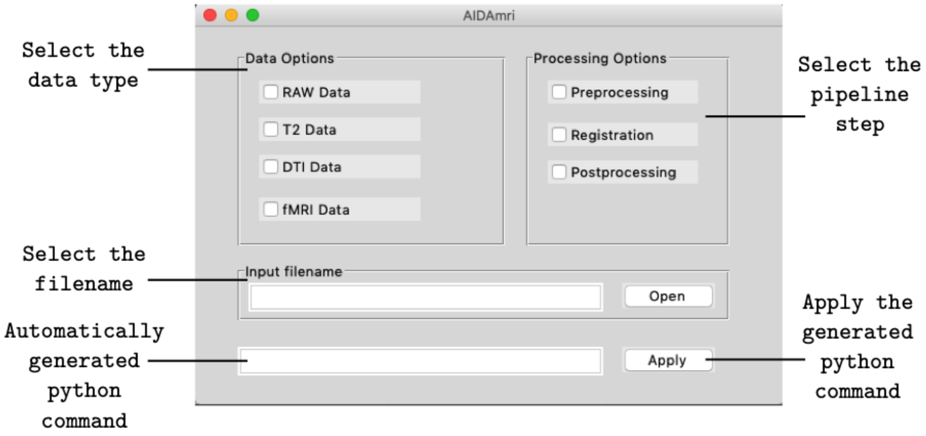

Figure 2: Description of the user interface to process the data step by step.

3.1 Convert raw data

Convert Bruker raw data to NIfTI files by specifying the folder containing all raw folders of each scan (see Figure 3). A file with exactly the same name is created in the given input folder. It contains all sorted NIfTI files. The raw data should have the same orientation as the example dataset.

```
python pv_conv2Nifti.py -i .../testData
```

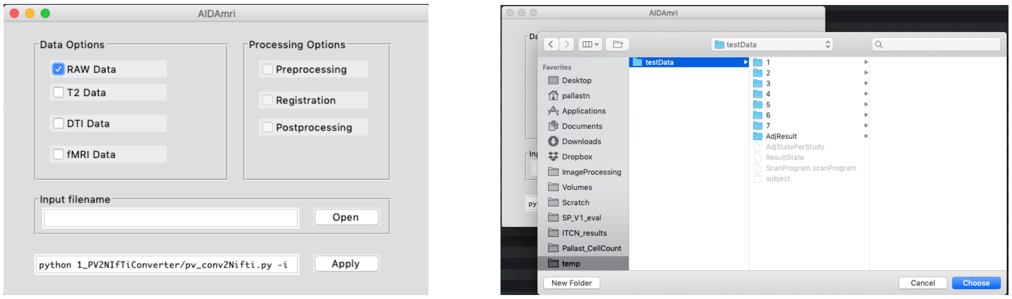

(a) Options and modes (b) Selected file

Figure 3: Convert raw data to NIfTI files by specifying the folder containing all raw folders of each scan

3.2 Processing of T2w & T2map data

Apply the reorientation, bias field correction and brain extraction to the T2w data set. The automatically attached endings of the processed filenames indicate which steps have been performed. Brain extraction should be of good quality and must be manually checked or corrected by adapting the default parameter (see Figure 4).

```
python preProcessing_T2.py -i .../testData/T2w/testData.5.1.nii.gz
```

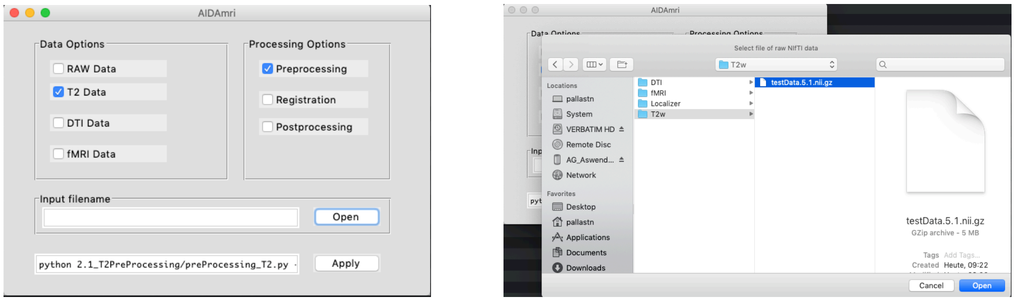

(a) Options and modes (b) Selected file

Figure 4: Apply the preprocessing to the T2 data by selecting the correct filename and options

The registration will also work without the following step. The user can segment a region by taking the brain extracted dataset as reference (ends with

...BET.nii.gz). We recommend to conduct this step with itk-SNAP. The saved file should end with the extension ...Stroke_mask.nii.gz

The next step includes the registration of the Allen Brain Reference Atlas with the brain extracted T2 dataset. The result is a variety of files. An impression of the registration can be obtained by superimposing the file the brain extracted file with the annotations of the Allen Brain (ends with ..._Anno.nii.gz) (see Figure 5)
`python registration_T2.py -i .../testData/T2w/testDataBiasBet.nii`

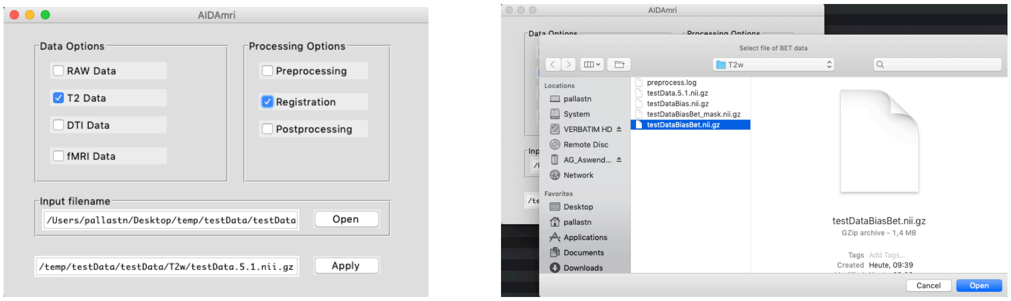

(a) Options and modes (b) Selected file

Figure 5: Apply the registration between the T2 data and the ARA by selecting the correct filename and options

If the user previously defined a region of interest, the region size, segmented parental ARA regions and segmented original ARA regions can be determined in that step. Here, the segmented region .../Stroke_mask.nii.gz is overplayed with the Allen Brain Reference Atlas and saved in the file ...Anno_mask.nii.gz. The user does not have to enter single files, but the path to the .../T2w folders (see Figure 6)

```
python getIncidenceSize_par.py -i .../testData/T2w
python getIncidenceSize.py -i .../testData/T2w
```

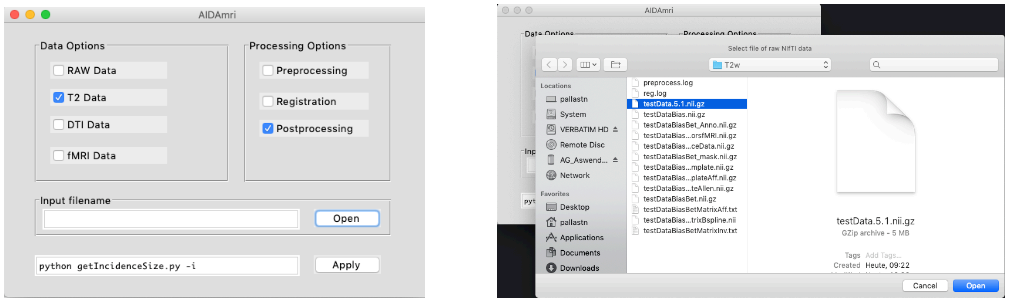

(a) Options and modes (b) Selected file

Figure 6: Get the previously defined region of interest, the region size, segmented parental regions and segmented original regions by selecting the correct filename and options

The results, such as affected regions ans ROI volume are stored in the folder `.../T2w` in the following files
`affectedRegions.txt`
`affectedRegions.nii.gz`
`affectedRegions_Parental.txt`
`affectedRegions_Parental.nii.gz`

3.3 Processing of T2 data

From the masks drawn on the T2-weighted images, it is possible to determine both the incidence map and the size of affective regions. For example, if a `day1` folder contains multiple `Mouse_1-Mouse_15` folders and the processed T2 data is in those folders, the command would be as follows

```
python getIncidenceMap.py -i .../day1 -s Mouse*
```

3.4 Processing of DTI data

The DTI processing procedure includes a dimension reduction, bias correction, a threshold application, and the subsequent brain extraction. The endings on the filenames indicate which steps have been performed (see Figure 7).

```
python preProcessing_DTI.py -i .../DTI/testData.7.1.nii.gz
```

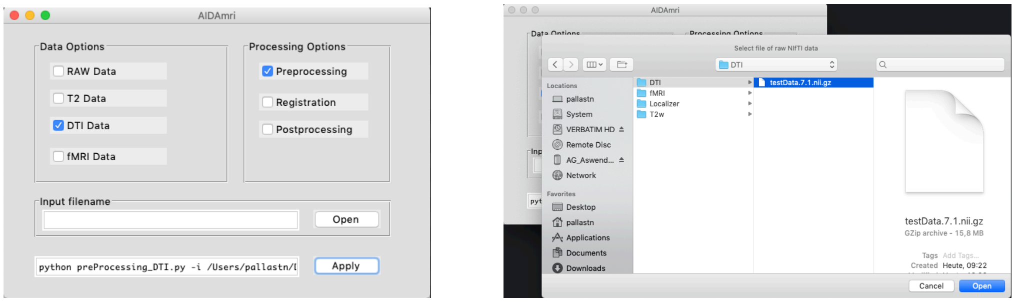

(a) Options and modes (b) Selected file

Figure 7: Apply the preprocessing to the DTI data by selecting the correct filename and options

The next step includes the registration of the Allen Brain Reference Atlas with the brain extracted DTI dataset. Here, two processing options are possible a) Registration of a reference mask that is related to an other dataset - append command `-r <filename of ref>` b) By omitting the command , the algorithm stroke mask from the same folder or no mask (see Figure 8).

`python registration_DTI.py -i .../DTI/testDataSmoothMicoBet.nii.gz`

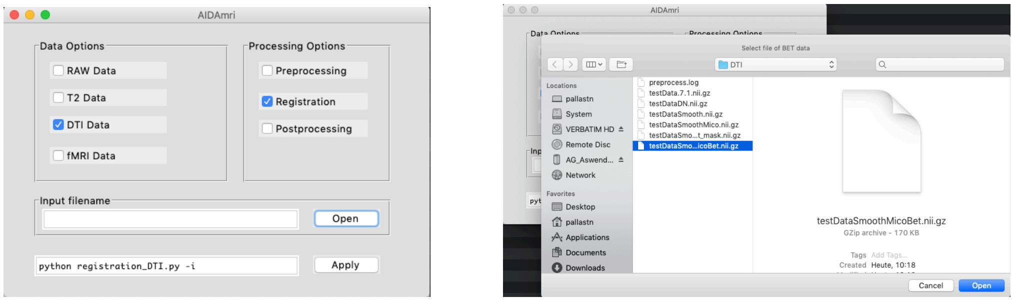

(a) Options and modes (b) Selected file

Figure 8: Apply the registration between the DTI data and the ARA by selecting the correct filename and options

The connectivity is finally calculated using DSI-Studio. All connectivity matrices are based on the reference atlas (see Figure 9).

`python dsi_main.py -i .../DTI/testData.7.1.nii.gz`

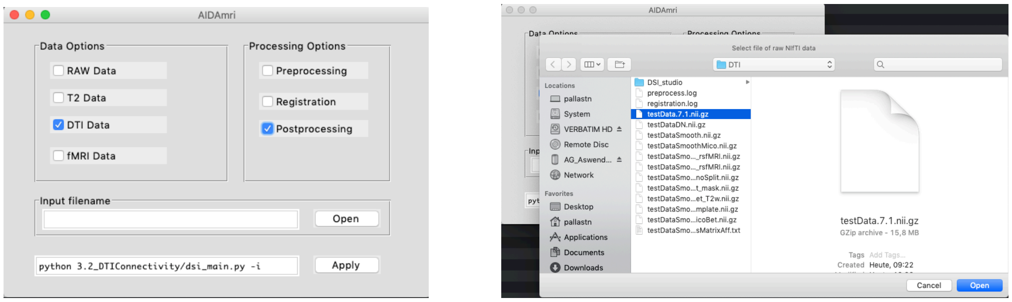

(a) Options and modes (b) Selected file

Figure 9: Process the DTI data with respect to the ARA regions by selecting the correct filename and options

The connectivity matrices of the parental ARA, the original ARA and the related ROI are stored in the folder `.../DTI/connectivity` as `.txt` and `.mat`. DSI-Studio differentiates between matrices that count how many pass and end in each region. The adjacency matrices can be visualised the related plot function.

```
python plotDTI_mat.py -i
.../testData/fMRI/connectivity/testData*.connectivity.mat
```

Processing of fMRI data

The fMRI processing is roughly comparable to the preprocessing of the DTI datasets. Brain extraction should be of good quality and must be manually checked or corrected by adapting the given parameters (see Figure 10).

```
python preProcessing_fMRI.py -i .../fMRI/testData.6.1.nii.gz
```

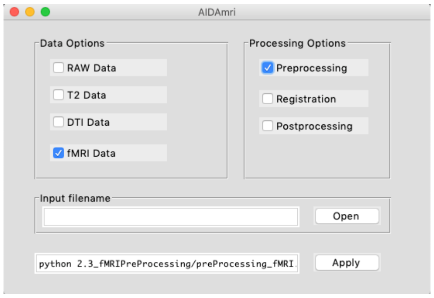

(a) Options and modes

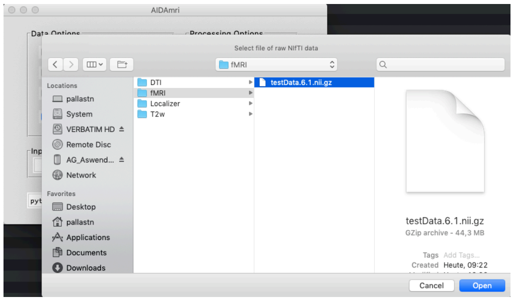

(b) Selected file

Figure 10: Apply the preprocessing to the rsfMRI data by selecting the correct filename and options

The step includes the registration of the Allen Brain Reference Atlas with the brain extracted fMRI dataset. The result is a variety of files. An impression of the registration can be obtained by superimposing the file the brain extracted file with the annotations of the Allen Brain (ends with `..._Anno.nii.gz`) (see Figure 11)
`python registration_fMRI.py -i .../testData/fMRI/testSmoothBet.nii`

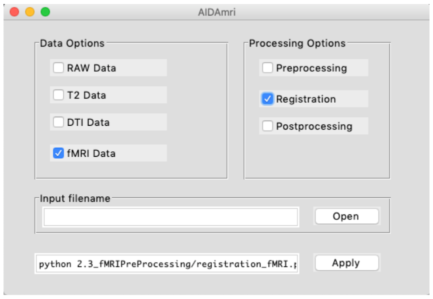

(a) Options and modes

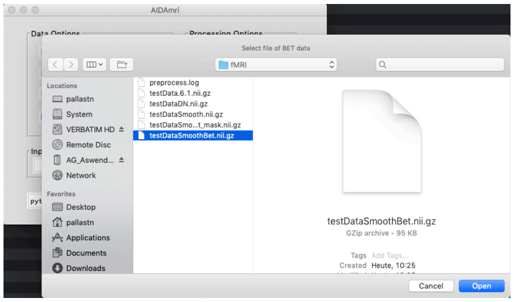

(b) Selected file

Figure 11: Apply the registration between the rsfMRI data and the ARA by selecting the correct filename and options

If physiological data are not available, the step will be conducted without the included regression. All activity matrices are based on the reference atlas (see Figure 12).
`python process_fMRI -i .../fMRI/testData.6.1.nii.gz`

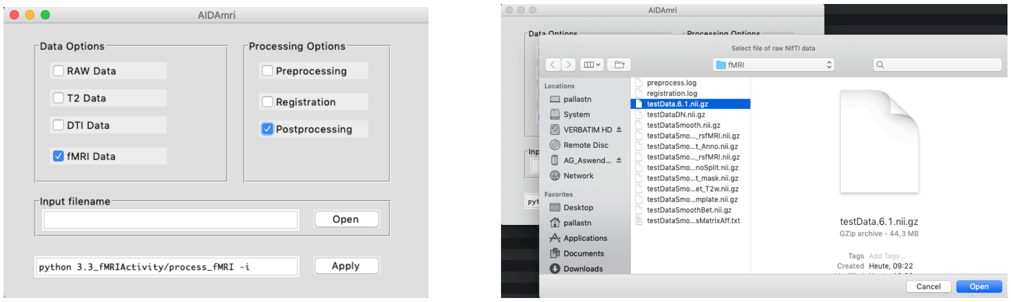

(a) Options and modes (b) Selected file

Figure 12: Process the rsfMRI data with respect to the ARA regions by selecting the correct filename and options

The activity matrices of the parental Atlas, the original Atlas are stored in the folder `.../fMRI/regr` as `.txt` and `.mat` with the prefix
`MasksTCs.` and `MasksTCsSplit.`.
The related adjacency matrices can be visualised the related plot function.
`python plotfMRI_mat.py -i .../testData/fMRI/regr/MasksTCsSplit*.mat`
